# Increased temporal sensitivity for threat: A Bayesian generalized linear mixed modeling approach

**DOI:** 10.3758/s13414-018-01637-9

**Published:** 2018-12-04

**Authors:** Jason Tipples

**Affiliations:** Psychology Group, School of Social, Psychological & Communication Sciences, Leeds Beckett University, Leeds, UK

**Keywords:** Temporal processing, Bayesian modeling

## Abstract

People overestimate the duration of threat-related facial expressions, and this effect increases with self-reported fearfulness (Tipples in *Emotion, 8,* 127–131, 2008, *Emotion, 11,* 74–80, 2011). One explanation (Cheng, Tipples, Narayanan, & Meck in *Timing and Time Perception, 4,* 99–122, 2016) for this effect is that emotion increases the rate at which temporal information accumulates. Here I tested whether increased overestimation for threat-related facial expressions in high fearfulness generalizes to pictures of threatening animals. A further goal was to illustrate the use of Bayesian generalized linear mixed modeling (GLMM) to gain more accurate estimates of temporal performance, including estimates of temporal sensitivity. Participants (*N* = 53) completed a temporal bisection task in which they judged the presentation duration for pictures of threatening animals (poised to attack) and nonthreatening animals. People overestimated the duration of threatening animals, and the effect increased with self-reported fearfulness. In support of increased accumulation of pacemaker ticks due to threat, temporal sensitivity was higher for threat than for nonthreat images. Analyses indicated that temporal sensitivity effects may have been absent in previous research because of the method used to calculate the index of temporal sensitivity. The benefits of using Bayesian GLMM are highlighted, and researchers are encouraged to use this method as the first option for analyzing temporal bisection data.

When faced with danger, people frequently report changes in their perception of time. In support of such reports, studies have repeatedly shown that people overestimate the duration of emotionally arousing images, such as faces of people expressing fear or anger (for reviews, see Droit-Volet, 2013; Lake, LaBar, & Meck, 2016). In keeping with the idea that this effect is related to emotional experience, studies have shown that the overestimation for threat-related expressions is increased in individuals with high levels of anxiety and self-reported fearfulness (Bar-Haim, Kerem, Lamy, & Zakay, 2010; Tipples, 2008, 2011). The novel goal of the present research is to extend my previous research that used faces (Tipples, 2008, 2011) by establishing whether individual differences in self-reported fearfulness might also moderate the overestimation effect for threat-related images. Previous studies have examined time estimation for threat-related images but have not tested for individual differences in fearfulness. Furthermore, I illustrate an alternative approach to analyzing temporal bisection data—namely, Bayesian generalized linear mixed modeling (GLMM). A key benefit of this approach is that it uses information shared across individuals to improve estimates of time perception, including estimates of temporal sensitivity.

A clear role for individual differences in time estimates for fear-related events has been reported in several studies. In one study (Tipples, 2011) that used the temporal bisection task, participants learned to recognize short and long durations in an initial training phase. Then, in a second phase, they were asked to indicate whether angry, fearful, and neutral facial expressions were displayed for a duration that was more similar to either a standard short or a long duration that they had learned earlier. The Weber ratio and bisection point were calculated as indices of temporal performance. The bisection point is the comparison duration giving rise to 50% of “long” responses. Relatively lower bisection point values indicate a perceived lengthening of time. The Weber ratio is an index of timing precision (or sensitivity) and is typically calculated by dividing the difference limen by the bisection point (e.g., Droit-Volet & Wearden, 2002; Ortega & López, 2008; Penney, Yim, & Ng, 2014). The difference limen is one half of the difference between the duration corresponding to a *p*(“long”) of 75% and the duration corresponding to a *p*(“long”) of 25%. The results showed that participants overestimated the duration of angry and fearful relative to neutral facial expressions—that is, the bisection point was reached sooner for the angry (*M* = 955 ms) and fearful (*M* = 951 ms) faces than for the neutral face (*M* = 1,021 ms). Moreover, this effect was modulated by individual differences in self-reported fearfulness. Specifically, fearfulness uniquely predicted increases in overestimation for both angry and fearful expressions. In keeping with other studies of emotion and time perception (e.g., Droit-Volet, Mermillod, Cocenas-Silva, & Gil, 2010), differences in temporal sensitivity indexed by Weber ratio values were not significant.

One explanation for the effects of facial expressions on time perception reported by Tipples (2011) is that emotional arousal increases the rate of a pacemaker mechanism that resides within an internal clock. Internal clock models of timing (Gibbon, 1977; Gibbon, Church, & Meck, 1984; Treisman, 1963; Treisman, Faulkner, Naish, & Brogan, 1990) include three key components: (1) a pacemaker that emits units of time (or pulses) at a specific rate, (2) an attention-controlled switch that controls the flow of the pulses, and (3) a counter through which perceived time is calculated on the basis of the total number of counted units. The switch closes when individuals attend to the to-be-stimulus and opens again when the stimulus offsets and timing finishes. A prediction of the internal clock model is that the effects of pacemaker speeding will multiply as duration increases—a multiplicative effect. In the temporal bisection task, a multiplicative pattern for emotion would be recorded if emotion leads to (1) a relative overestimation of time and (2) an increase in the gradient of the slope for duration (Cheng, Tipples, Narayanan, & Meck, 2016). Although Tipples (2011) did report an overestimation effect, the differences in temporal sensitivity were not significant, and more broadly, a multiplicative pattern has not been consistently found for emotionally arousing stimuli, including threat-related expressions (for a review, see Lake et al., 2016).

The first goal of the present work was to extend the study by Tipples (2011) by establishing whether the association between individual differences in fearfulness and overestimation of time for threat generalizes to pictures of threatening animals. Other studies (Grommet et al., 2011) have reported an overestimation effect for fear-related animals but have not tested for individual differences in fearfulness. Here I specifically selected images that depict threat toward the observer (e.g., a shark with exposed fangs) in an attempt to produce a clear effect of threat on the time estimates.

The second goal of this research was to illustrate the benefits of the Bayesian GLMM approach for analyzing temporal bisection data, and in particular, the benefits of Bayesian GLMM for estimating differences in the psychometric slope for duration between threat and neutral conditions. Tutorials and software for fitting non-Bayesian GLMMs to psychophysical data can be found in several recent publications (e.g., Knoblauch & Maloney, 2012, chap. 9; Moscatelli, Mezzetti, & Lacquaniti, 2012). GLMMs extend the more usual approach to modeling temporal bisection data—a generalized linear model with either a probit or logit link function—to include both information pooled across individuals and repeated measures from the same individual. Pooling information across individuals is desirable because it permits shrinkage (Efron & Morris, 1977; Stein, 1956), whereby the distribution of individual parameters (the slope and intercept of the psychometric functions) are pulled toward the group mean relative to the standard “no-pooling” approach, in which information is not shared across individuals.

Pooling information is likely to be particularly useful in studies of emotion and time perception, in which the number of repetitions per cell of the design is typically low (< 10), and consequently, the data are particularly prone to complete separation (i.e., all short durations being classified as short and all long durations being classified as long). Without pooling, each psychometric function is weighted equally, and consequently, individual very steep or very shallow psychometric functions can exert undue influence on estimates of the average psychometric function. In support of the use of GLMMs for temporal bisection data specifically, a simulation study (Moscatelli et al., 2012) showed that GLMMs have higher statistical power than the more traditional “no-pooling” approach (e.g., Tipples, 2011), in which individual slopes and intercepts are calculated for everyone separately and then used in a separate statistical analysis.

There are several reasons to use a Bayesian as compared to a non-Bayesian GLMM. First, a Bayesian approach can provide researchers with answers to questions they typically want—the probabilities of specific hypotheses. For example, the conclusion that threat does not affect temporal sensitivity is based on nonsignificant *p* values. However, a nonsignificant *p* value does not provide support for the null hypothesis, but rather for the probability of these, or more extreme, data under the null, *p*(*D* | H_0_). Bayesian methods are more suited to drawing the conclusion that a difference is practically equivalent to zero (or another value). A second benefit of using Bayesian GLMMs, specifically, is that maximum-likelihood estimates of the variance–covariance matrix estimated from frequentist GLMMs are sometimes drawn toward upper or lower boundary values (Bolker, n.d.)—in such situations, Bayesian methods avoid boundaries by providing a posterior distribution of values rather than a point estimate (Chung, Rabe-Hesketh, Dorie, Gelman, & Liu, 2013).

In summary, the goals were (1) to extend both Tipples (2011) and Grommet et al. (2011) by establishing whether the association between individual differences in fearfulness and overestimation of time for threat generalizes to pictures of threatening animals, and (2) to illustrate the benefits of the Bayesian GLMM approach for analyzing temporal bisection data.

## Method

### Sample size and power

In previous research (Tipples, 2011), I recorded a relatively large zero-order correlation (*r* = .51) between an index of the overestimation effect for threat faces (*z*-score-transformed proportion of “long” responses for threat-related minus *z*-score-transformed proportion of “long” responses for neutral faces) and reported fearfulness. In a frequentist setting, only 22 participants are needed to achieve 80% power (alpha = .05, one-tailed). Nonetheless, in previous research the correlation dropped to *r* = .38 after controlling for other covariates, and therefore I planned to sample as many participants as possible (within a single semester) on the condition that I sampled at least 44 participants—sufficient participants to achieve 80% power (alpha = .05, one-tailed) for an effect size as small as *r* = .38. The final sample size was 53 participants. The raw data, codebook, and code for the analyses can be found online https://osf.io/zyax5/.

### Participants

Fifty-three undergraduate psychology students (29 female, mean age = 23.92 years, *SD* = 8.46; 24 male, mean age = 22.31 years, *SD* = 5.07) from the University of Hull participated in return for a course credit. All had normal or corrected-to-normal vision.

### Stimuli and apparatus

In the testing phase, 14 pictures from the IAPS (Lang, Bradley, & Cuthbert, 2008) measuring 25.8 cm wide and 19.2 cm high were presented in the center of the computer monitor. Seven of the pictures were selected as dangerous animals, poised to attack (three snakes, one bear, two sharks, and one attacking dog), and seven as neutral animals (gannet, dog, hawk, butterfly, bird, coyote, and cow). The images were rated in separate research (Lang et al., 2008) on 9-point scales designed to measure their affective valence (ranging from *pleasant* to *unpleasant*) and arousal (ranging from *calm* to *excited*) experienced when viewing the images. The threat-related animals were rated as being more arousing (*M* = 6.58) and less pleasant (*M* = 3.74) than the neutral animals (arousal *M* = 4.00, valence *M* = 6.33). All stimuli were presented on a 17-in. computer monitor (1,280 × 1,080, 60 Hz) connected to a 1-GHz Pentium computer. Stimulus presentation and data collection were controlled by the E-Prime software (Schneider, Eschman, & Zuccolotto, 2002).

### Procedure

All participants completed learning and test phases. In the learning phase, participants were trained to discriminate short (400-ms) from long (1,600-ms) stimulus durations. On the first eight trials, a pink oval appeared for either a short or a long duration in a fixed sequence (e.g., long–short–long–short–. . .). Participants were told to expect this sequence and to press the “z” or “m” key to indicate whether the oval appeared for a short or a long duration. The response mapping (e.g., “z” for short durations and “m” for long durations) was counterbalanced across participants. Following a response, participants were presented with visual feedback lasting 500 ms, for both correct (“yes”) and incorrect (“no”) decisions. The feedback was followed by a fixed 1,000-ms intertrial interval. In the final stage of the learning phase, the pink oval was presented for a further eight trials in a new random order for each participant. Participants continued to indicate whether the oval appeared for a short or a long stimulus duration and received feedback.

During the test phase, the oval was replaced on each trial by one of the 14 different images, displayed for one of seven durations. Participants were asked to (a) look at the picture and (b) indicate whether the picture appeared for a duration that was closer to either the short or the long duration that they had learned earlier. Feedback was not given during the main test phase. In the test phase, 14 possible trial types were derived from the factorial combination of Duration (7) × Picture Type (2; threat, neutral). Each of the 14 trial types was repeated seven times, leading to the creation of 98 trials for each person. Each trial began with a fixed 800-ms blank interval before the onset of a picture stimulus. A new randomized trial order was created for each participant. Finally, after the main test phase, participants completed the Emotionality, Activity and Sociability Temperament Survey for Adults (EAS; Buss & Plomin, 1984) and the State–Trait Anxiety Inventory–Trait Form Trait subscale (Spielberger, Gorsuch, Lushene, Vagg, & Jacobs, 1983).

## Results

### Traditional method

To estimate a psychometric curve for each person for each picture type, I modeled the number of “long” responses using a binomial generalized linear model (GLM) with a logistic link function in R (R Core Team, 2018). Nagelkerke’s pseudo R-squared was used as an index of model fit. Boxplots of the pseudo-R-squared values showed that the data of one participant were more than 3 × the interquartile range below the first quartile for the sample (pseudo *R*-squared = .21), and therefore the data for this individual were removed prior to the statistical analyses. For the remaining 52 participants, the bisection point (BP), difference limen (DL), and Weber ratio (WR) were calculated for each participant and each picture type, separately. I calculated the WR in two ways: (1) by dividing the DL by the BP (e.g., Droit-Volet & Wearden, 2002; Ortega & López, 2008; Penney et al., 2014) and (2) by dividing the DL by the arithmetic mean of the short and long durations (Mioni et al., 2018). The mean Nagelkerke’s pseudo-R-squared values indicated overall satisfactory fits for both the neutral (*M* = .97, *SD* = .05) and threat (*M* = .98, *SD* = .01) image conditions.

The mean BP and both WR indices for each participant were analyzed using paired *t* tests. Following previous research (Grommet et al., 2011) that had also used threat-related images, the estimated BP was reached sooner for threat (BP mean = 954 ms, *SD* = 181) than for neutral (BP mean = 1,009 ms, *SD* = 181) images, *t*(51) = – 2.71, *p* = *.*009 (95% CI [– 95, – 14]); that is, participants needed more temporal information in order to judge a neutral picture as long. For the WR calculated by dividing the DL by the BP, the results showed that for the difference between threat (WR mean = .168, *SD* = .08) and neutral (WR mean = .195, *SD* = .17), the effect was not significant, *t*(51) = – 1.29, *p* = *.*20 (95% CrI [– 0.06, 0.01]). However, analyses of the WR calculated by dividing the DL by the arithmetic mean of the short and long durations (1,000 ms) showed a significantly lower WR for threat (WR mean = .157, *SD* = .07) than for neutral (WR mean = .187, *SD* = .09) images, *t*(51) = -2.44, *p* = *.*01 (95% CI [– 0.05, – 0.005]). Similarly, the DL was lower for threat (DL mean = 157, *SD* = 76.9) than for neutral (DL mean = 187, *SD* = 98.5) images, *t*(51) = – 2.48, *p* = *.*01 (95% CI [54.14, 5.36]).

In summary, replicating previous research, there was a significant, large 55-ms leftward shift in the BP, indicating overestimation for threat as compared to neutral images. Temporal sensitivity increased for threat as compared to neutral images, but only when the index of temporal sensitivity was calculated by dividing the DL by the arithmetic mean, and not when using the more typical method of dividing the DL by the BP.

Following previous research, self-reported fearfulness was measured by scores on the Fearfulness subscale of the EAS (Buss & Plomin, 1984), and trait anxiety was measured using the State–Trait Anxiety Inventory–Trait Form (STAI-T; Spielberger et al., 1983). Scores on each of the subscales of the EAS and STAI-T are presented in Table 1. The mean fearfulness score for the sample (8.98, *SD* = 3.58) was similar to the mean score (9.28, *SD* = 3.68) reported in previous research (Tipples, 2011). Independent *t* tests with participant sex as the between-subjects variable and each measure shown in Table 1 as the dependent variable indicated that (1) mean fearfulness scores were higher for female (*M* = 9.86) than for male (*M* = 7.92) participants, *t*(51) = 2.01, *p* = .049 (95% CrI[0.002, 3.88]), and (2) all other differences were nonsignificant (all *t* values < 1.05, all *p* values > .3).

**Table 1:** Individual differences: Means and standard deviations of scores for male and female participants, separately, for each subscale of the Emotionality, Activity and Sociability (EAS) Temperament Survey for Adults (Anger, Distress, Fearfulness, Sociability, Activity), the State–Trait Anxiety Inventory–Trait Form, Trait subscale (STAI-T; Spielberger, Gorsuch, Lushene, Vagg, & Jacobs, 1983), and age

Measure	Males (*N* = 24)		Females (*N* = 29)	
	*M*	*SD*	*M*	*SD*
EAS–Activity	9.79	3.85	10.04	3.67
EAS–Anger	9.93	3.58	10.33	3.51
EAS–Distress	9.79	3.65	8.79	3.37
EAS–Fearfulness	7.92	3.55	9.86	3.35
EAS–Sociability	12.38	3.32	12.38	3.36
STAI-T	43.24	9.32	43.50	9.59
Age	22.31	5.07	23.92	8.46

### Bayesian GLMM

The data were modeled in a Bayesian GLMM in which the number of “long” responses was modeled as a binomially distributed random variable with a logistic link function. Modeling was carried out using the brms package (Bürkner, 2017) as an interface between Stan (Carpenter et al., 2017) and R (R Core Team, 2018). In the Bayesian approach, plausible values of a model parameter—for instance, likely values of the slope representing increased sensitivity due to fear—are proportional to the likelihood of the data (conditioned on the model parameters) multiplied by priors for the parameters. The Bayesian modeling approach uses Markov chain Monte Carlo (MCMC) sampling to estimate a range of probable values for the model parameters. MCMC requires checks for chain convergence. To illustrate convergence, chain convergence diagnostics for the Duration × Image interaction effect (for Model 4 described below) can be found in the online supplementary material (https://osf.io/zyax5/). Also in the online supplementary material, I have included a graphical posterior predictive check to assess the adequacy of the main model, as well as the code for the analyses. The data set ("timeDF.csv") is also avaliable (https://osf.io/zyax5/). Weakly informative priors were selected for all model parameters.

### Model specification

I tested a single model that included the Image Type × Duration × Fearfulness interaction term. The model included varying by-subject intercepts and varying by-subject slopes (for image type and duration), as well as a correlation coefficient between the varying by-subject intercepts and slopes. Variance by items (for each specific image) could not be estimated, due to a coding error. In the regression equation, image type (neutral, threat) was entered as a categorical (treatment-coded) input variable, with the neutral image condition serving as the baseline (“0”) and threat serving as the treatment (“1”). The continuous variable duration was mean-centered—that is, 1,000 ms was subtracted from each of the duration conditions—and fearfulness scores were both mean-centered and scaled to two standard deviations (Gelman, 2008). Mean-centering facilitates the interpretation of regression coefficients and also improves MCMC sampling efficiency.

**Fig. 1:**
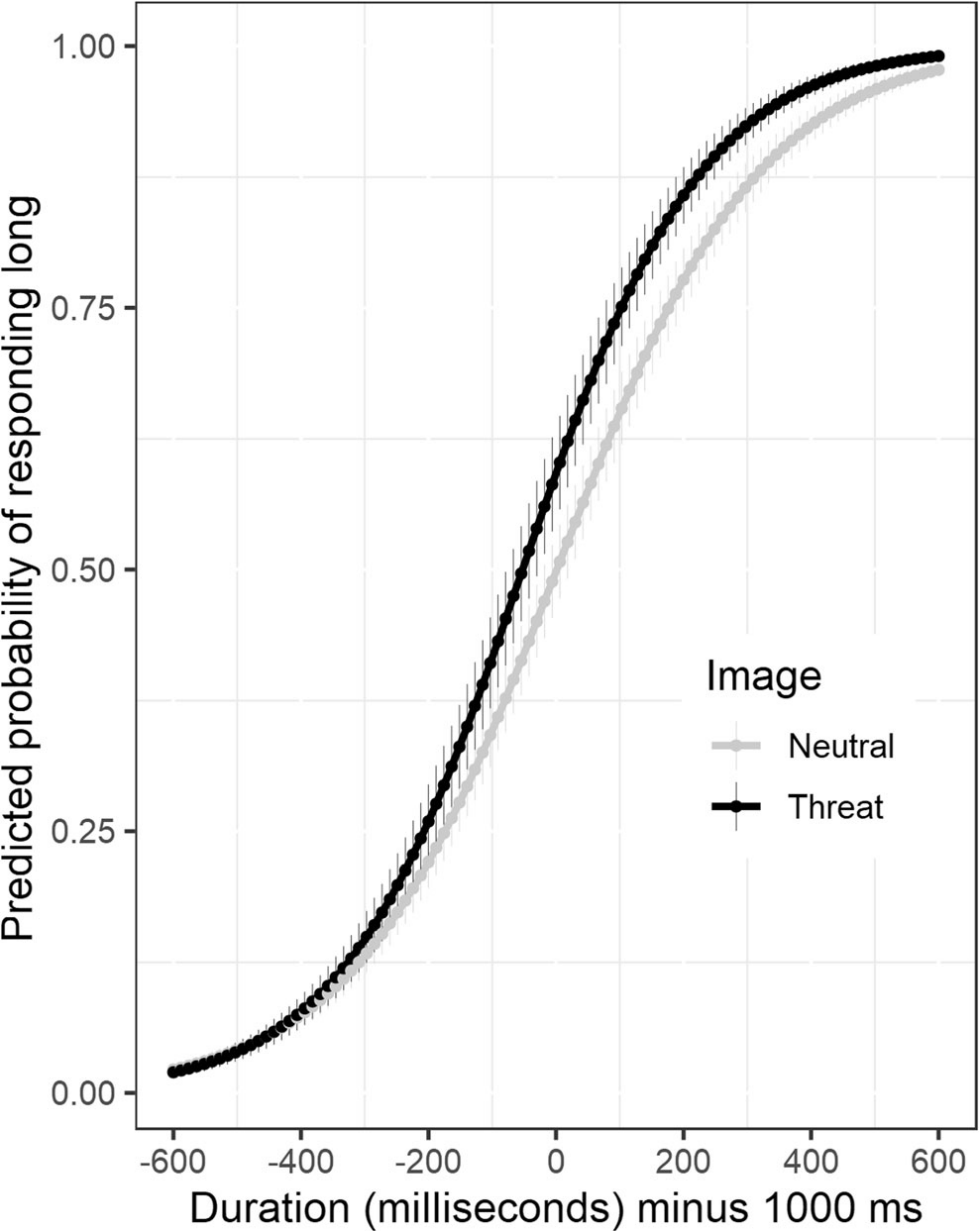
Multilevel logistic regression model of the proportions of “long” responses, showing average model-predicted probabilities as a function of image (neutral, threat) and duration (mean-centered by subtracting 1,000 ms)

As is shown in Fig. 1, at mean levels of fearfulness, the estimated slope for duration was steeper for threat-related than for neutral animals, *β*(Threat × Duration) = 0.0008, 95% CrI [0.0001, 0.0016]. In other words, the model results agree with the analyses reported above (in which I calculated the WR by dividing the DL by the arithmetic mean): Temporal sensitivity increased for threat as compared to neutral images. There was a small decrease in the duration slope for threat images with increases in levels of fearfulness, although the 95% credible interval for this effect included zero, *β*(Duration × Threat × Fearfulness) = – 0.0004 (95% CrI[– 0.0016, 0.0008]).

**Fig. 2:**
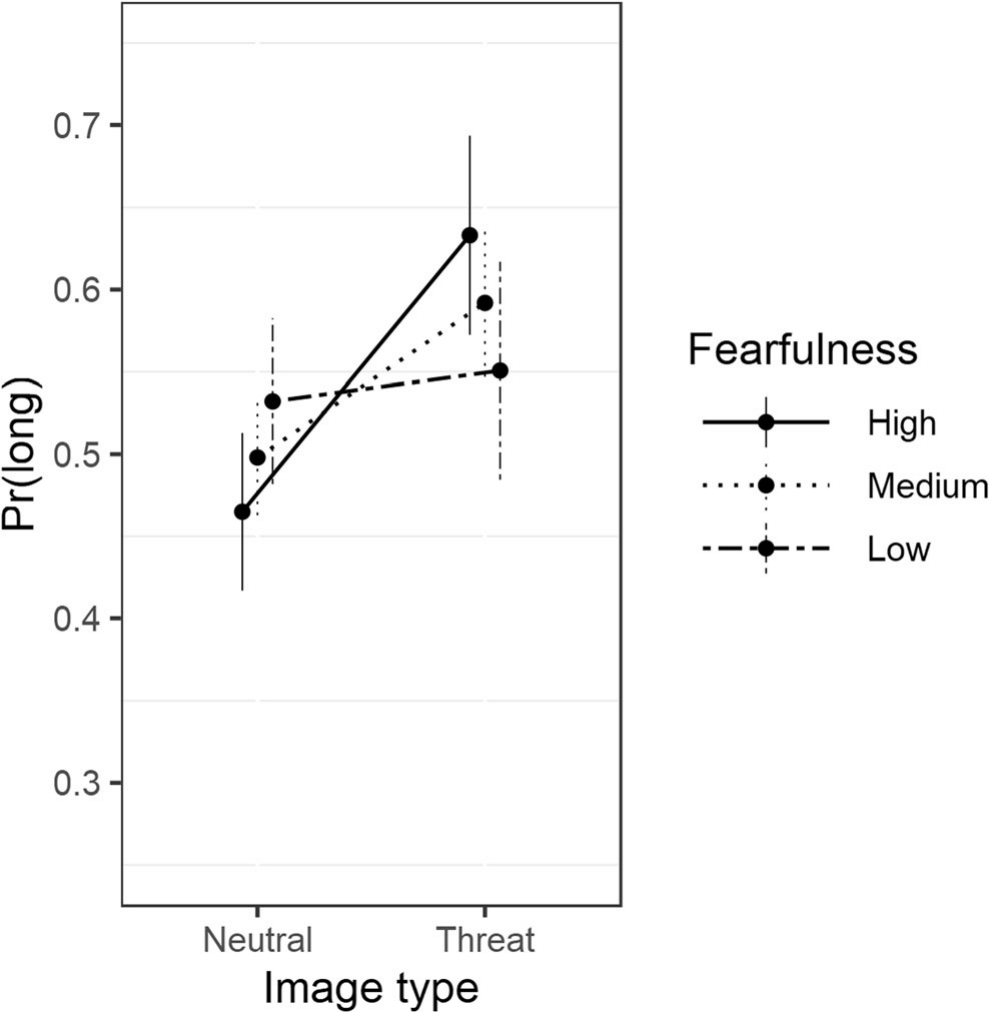
Multilevel logistic regression model of the proportions of “long” responses, showing average model-predicted probabilities of responding “long” as a function of image (neutral, threat) and fearfulness (low, medium, high). Error bars show standard errors of the predicted responses

Other results corroborate the findings reported in previous research. Responding “long” increased for threat images relative to neutral images, *β*(Threat) = 0.3898 (95% CrI[0.1495, 0.6368]), and moreover, this effect increased with self-reported fearfulness, *β*(Threat × Fearfulness) = 0.6213 (95% CrI[0.1522, 1.091]). The latter effect is shown in Fig. 2, where it can be seen that at the average duration, the overestimation effect for threat-related images increased with levels of fearfulness from one *SD* below the mean (low fearfulness) to one *SD* above the mean (high fearfulness). Taking the exponent of the log-odds and converting the odds to a probability [*p*/(1 – *p*)] helps us understand these results. At mean levels of fearfulness, the mean of the posterior probability of responding “long” at the mean (1,000-ms) duration for the neutral image condition is .49 (BP = 1,001 ms). The 95% credible interval for the latter effect includes .50 [.42, .56], and therefore we can conclude that participants with medium levels of self-reported fearfulness were relatively accurate with respect to clock time. The mean posterior probability of responding “long” increased to .59 (BP = 946 ms) for threat images, and the latter effect increased further to *p* = .62 (BP = 889 ms) for individuals with high levels of self-reported fearfulness. In short, the results support both relative and absolute (as compared to clock time) overestimations for threat-related images.

In addition to the main model, I estimated a final model that showed that the Fearfulness × Image Type interaction continued to exclude zero, *β*(Threat × Fearfulness) = 0.80 (95% CrI [0.10, 1.49]), after controlling for the relationships between image (neutral, threat) differences and mean-centered scores for the remaining individual differences: *β*(Threat × Sociability) = 0.15 (95% CrI [– 0.35, 0.67]); *β*(Threat × Activity) = – 0.23 (95% CrI [– 0.78 , 0.29]); *β*(Threat × Anger) = – 0.08 (95% CrI [– 0.60, 0.41]); *β*(Threat × Distress) = 0.42 (95% CrI [– 0.28 , 1.13]); *β*(Image × STAI) = – 0.68 (95% CrI [– 1.37, 0.007]).

### Shrinkage

**Fig. 3:**
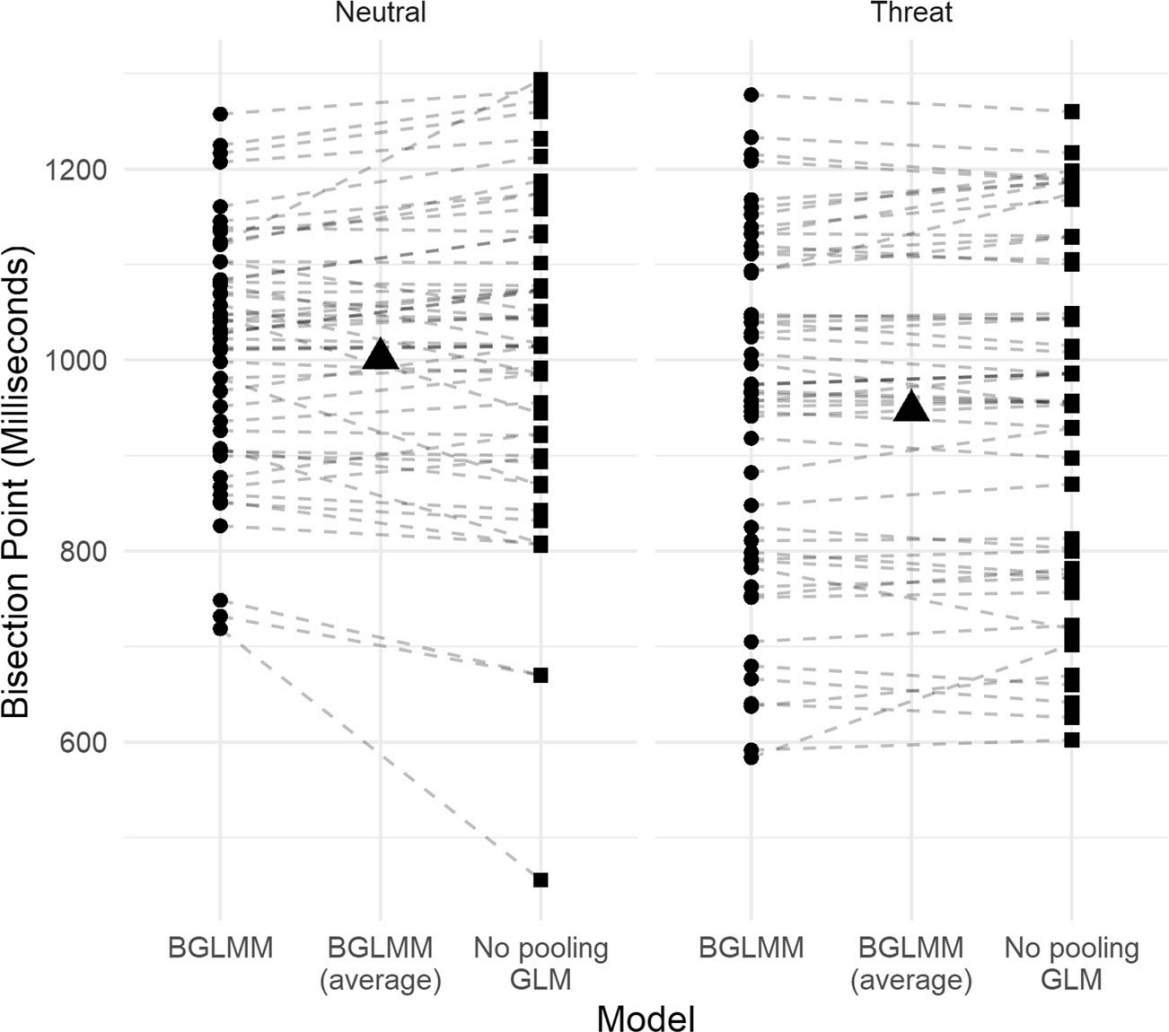
Participant bisection points for neutral and threat animals estimated from a Bayesian GLMM and a no-pooling GLM (traditional approach). The black triangles represent the group-average bisection points from the Bayesian GLMM. Shrinkage—that is, movement toward the group-averaged posterior estimate of the bisection point—can be seen most clearly for the lowest three no-pooling estimates for neutral images

To illustrate shrinkage, whereby individual regression coefficients are pulled toward the group mean, I have plotted (Fig. 3) the BPs for neutral and threat images for each individual separately, calculated from the Bayesian GLMM and the traditional, no-pooling GLM. To allow for direct comparisons between the Bayesian GLMM and the no-pooling GLM estimates, I reestimated the Bayesian GLMM without fearfulness scores. The black triangle represents the group-average BP from the Bayesian GLMM. Perhaps the most striking aspect of this plot is the considerable heterogeneity in the BPs—there are very clear individual differences. Shrinkage can be seen most clearly for the neutral images, where three very low BPs are pulled toward the group average (represented by the triangle in the center of the left plot).

### Exploratory analyses

I ran an exploratory analysis to test whether fearfulness was associated with time perception for threat in the high fearfulness-scoring female participant group. The regression model was identical to the main model described above but used the data for female participants only. For female participants, responding “long” increased for threat images relative to neutral images, *β*(Threat) = 0.61 (95% CrI[0.30, 0.94]), and moreover, this effect increased with self-reported fearfulness, *β*(Threat × Fearfulness) = 0.61 (94% CrI[0.01, 1.2]). In other words, individual differences in fearfulness are associated with the overestimation effect for threat within the (high fearfulness-scoring) female participant group.

## Discussion

This study extended the research by showing that the previously reported overestimation effect for threatening facial expressions in high-fearful individuals (Tipples, 2011) generalizes to images of threatening animals poised to attack. The effects are not restricted to one class of stimuli. A new finding is that at medium levels of fearfulness, temporal sensitivity increased for threatening images. Increased temporal sensitivity was evident in a steeper slope both for duration and, relatedly, for the DL—the minimum observable difference in durations that produces a change in temporal judgments. This provides direct support for the pacemaker-speeding hypothesis (Cheng et al., 2016, p. 105) : “All things being equal, a faster clock should be a more accurate clock, thereby leading to improved sensitivity to time and lower difference limens.” The results also support the idea that threat-related stimuli engage fear-specific processes (Tipples, 2011), because the general overestimation effect for threatening images increased with self-reported levels of fearfulness, and the effect remained after controlling for the association between time estimates and other self-reported individual differences (in anxiety, sociability, distress, anger, and activity). The latter result extends those from a previous study (Tipples, 2011) that had used threat-related facial expressions, by showing that the effect generalizes to time estimates for threatening animals (poised to attack).

Considering the differences in temporal sensitivity reported here, I reanalyzed data from my previous study (Tipples, 2011), in which I examined time perception for angry, fearful, and neutral facial expressions. In that study I had reported nonsignificant differences in temporal sensitivity (indexed by the WR) between angry, fearful, and neutral facial expressions. Here I estimated a single Bayesian GLMM that included both the *β*(Duration × Fearfulness) and *β*(Duration × Anger) interaction terms, with the neutral face condition serving as a baseline (intercept) and duration as a mean-centered input variable. Convergence diagnostics and a table of the group-level posterior estimates can be found in the supplementary material (https://osf.io/zyax5/). In keeping with the present results for threat images, the estimated slope for duration was steeper for the fearful than for the neutral faces, *β*(Duration × Fearfulness) = 0.001, 95% CrI[0.0003, 0.001], and also steeper for the angry than for the neutral faces, *β*(Duration × Anger) = 0.0007, 95% CrI[0.00003, 0.001]. In sum, reanalyses of previous research (Tipples, 2011) corroborate the findings reported here—increased temporal sensitivity for threat.

A practical implication of this is that individual differences in fearfulness are consistently associated with an increase in the temporal overestimation effect for threat-related stimuli, and therefore, researchers may wish to include self-reported measures when modeling the effects of threat on time perception. The Bayesian GLMM reported here is a particularly attractive method to model such differences, because individual difference scores can simply be added to the model as a continuous covariate. Interaction effects in regression have a different interpretation than interaction terms in standard (sum-coded) analysis of variance, and therefore researchers not familiar with interpreting interaction terms in regression may wish to consult tutorials (e.g., Jaccard, 2001) on this topic. As I have demonstrated for this dataset, there are other benefits of Bayesian GLMM—namely shrinkage, whereby extreme values are shrunk toward the group mean, and consequently such values do not exert an undue influence on estimates of the regression coefficients. To facilitate the continued use of this modeling technique, I have posted the code for the analyses here at https://osf.io/zyax5/.

The findings do not support the conclusion (Droit-Volet, 2013) that threat does not affect temporal sensitivity. As these analyses illustrate, steeper slopes for threat-related stimuli may have been absent in previous research because of the method used to calculate the Weber ratio as an index of gradient of psychometric slope. In the present study, slope differences between threat and neutral images were not significant when the index of the gradient of the WR was calculated by dividing the difference limen by the bisection point. In contrast, calculating the WR by dividing by the average of the durations (1,000 ms) produced a result consistent with (1) estimates of the slope and intercept from the Bayesian GLMM analyses (slopes for threatening as compared to neutral animals) and (2) differences in the DL. For future research, I recommend calculating the WR by dividing by the arithmetic or geometric mean. In short, the results reported here are indicative of increased temporal sensitivity for threat, and therefore are consistent with the pacemaker-speeding account (Cheng et al., 2016).

A further reason that differences in temporal sensitivity might be absent in other studies of the effects of threat on time perception is that the psychometric functions calculated for temporal bisection data are subject to restriction-of-range effects. For example, if participants start responding “long” sooner for threat-related stimuli, their intercept will be higher on the *y*-axis, and consequently they might reach an upper limit (ceiling) sooner. Indeed, this is the exact pattern reported in one study (see Fig. 1 in Droit-Volet et al., 2010), in which the authors reported an overestimation of the duration of a cue signaling an aversive sound as compared to a nonaversive cue, but no differences in temporal sensitivity. Here the estimated that the (mean-centered) intercept for participants at medium levels of fearfulness was close to the average duration, and therefore the slopes for these participants were not subject to restriction-of-range effects. As is illustrated in Fig. 1, the estimated probability of responding “long” was close to zero for the shortest duration. For participants with high levels of self-reported fearfulness, the intercept was shifted upward, and in keeping with the restriction-of-range idea, there was a small decrease in the duration slope for threat images with increases in levels of fearfulness, albeit a difference that included zero. Therefore, modeling individual differences has a further benefit—it can highlight upward shifts in the intercept that might obscure the recording of differences in the gradient of psychometric functions.

One shortcoming of the present study is that only fear- or threat-related images were used, and therefore we cannot be sure to what extent the temporal sensitivity effects generalize to other emotional stimuli. One possibility is that the effects are specific to threat-related stimuli and reflect an adaptive, preparation-for-action response. Put differently, increased temporal sensitivity might help individuals be better prepared to respond to threat. Therefore, increased temporal sensitivity may not hold for other emotionally arousing images for which heightened accuracy is not needed. For example, increased temporal sensitivity might not be adaptive when viewing disgust-related (Grondin, Laflamme, & Gontier, 2014) or pleasant erotic (Angrilli, Cherubini, Pavese, & Mantredini, 1997) images. Future research will be necessary to test this idea.

In summary, the results extend previous research by showing that the overestimation effect previously reported for threatening animals increases in magnitude in individuals who report high levels of fear. In addition, I have shown that increased temporal sensitivity due to threat may have been absent in previous research due to the method used to calculate an index of the slope of the psychometric function. Finally, I have demonstrated the usefulness of the Bayesian GLMM approach to modeling temporal bisection data, and in particular how modeling shrinkage by pooling information across individuals can improve estimates of the location and gradient of the psychometric function.
